
JOURNAL INFORMATION
==============================
NLM Title Abbreviation: ScientificWorldJournal
Journal ID: ScientificWorldJournal
NLM Journal ID: 101131163
Journal ID: TSWJ

The Scientific World Journal

ISSN: 2356-6140
EISSN: 1537-744X
Publisher: Wiley

ARTICLE INFORMATION
==============================
PMCID: PMC6009240
PMID: 29973809
DOI: 10.1100/tsw.2002.35
Article ID: 312407
Article version: 1
Subjects: Short Report

Application of In Vitro Protein Expression to Human Prote

Beernink Peter T. 1 *
Krupka Simone S. 1
Lao Victoria 1
Martin George 2
Coleman Matthew A. 1
Molecular & Structural Biology Division, Lawrence Livermore National Laboratory, 7000 East Ave., Livermore, CA 94550;, USA
Roche Diagnostics Corp., 2929 7th St., Ste. 100, Berkeley, CA 94710, USA
*Peter T. Beernink: ptb@llnl.gov
Publication date: 2002
Electronic publication date: 2002 May 21
Volume: 2
First page: 73
Last page: 74
Copyright: Copyright © 2002 Peter T. Beernink et al.
Copyright year: 2002
License: This is an open access article distributed under the Creative Commons Attribution License, which permits unrestricted use, distribution, and reproduction in any medium, provided the original work is properly cited.
License URL: https://creativecommons.org/licenses/by/3.0/

Funding: Lawrence Livermore National Laboratory, Office of Science W-7405-Eng-48

==============================
Advances in Gene Technology: The Genome and Beyond –
Structural Biology for Medicine (Proceedings of the 2002 Miami
*Nature Biotechnology* Winter Symposium)
*TheScientificWorld* 2002, 2(S2), 73–74
ISSN 1532-2246; DOI 10.1100/tsw.2002.35

### APPLICATION OF *IN VITRO* PROTEIN EXPRESSION TO HUMAN PROTEOMICS

Peter T. Beernink[1], Simone S. Krupka[1], Victoria Lao[1], George Martin[2], and Matthew A. Coleman[1]
[1]Molecular & Structural Biology Division, Lawrence Livermore National Laboratory, 7000 East Ave., Livermore, CA 94550; [2]Roche Diagnostics Corp., 2929 7th St., Ste. 100, Berkeley, CA 94710
*ptb@llnl.gov

**INTRODUCTION**. A critical step in many proteomics projects is the identification of genes that express proteins in sufficient quantities for subsequent studies. LLNL houses a collection of >200,000 human cDNAs, known as the IMAGE Consortium (http://image.llnl.gov)[1], including some full-length enriched cDNA libraries. We have developed an integrated system that exploits coupled *in vitro* transcription/translation (IVT) to identify human IMAGE cDNAs that express proteins of the predicted mass. Moreover, this expression screening can be conducted without plasmid DNA preparation or subcloning. IVT-produced proteins are used directly in functional assays or the cDNAs are transported to a bacterial expression system for large-scale protein production.

**METHODS**. IVT reactions are performed using the RTS-500 Rapid Translation System (Roche Molecular Biochemicals) in conjunction with a biotin-lysine-tRNA conjugate, FluoroTect™ Green$_{Lys}$ (Promega). IVT products are visualized on a UV transilluminator or quantitated on a Packard fluorescence plate reader ($\lambda_{ex}$ = 485 nm; $\lambda_{em}$ = 510 nm). For micro- and far-western blots, 100 nl of IVT-produced protein are spotted on a glass slide. Detection is accomplished using adapted protocols and a rhodamine-conjugated secondary antibody. Crystallization experiments are conducted by the LLNL TB Crystallization Center (http://www-structure.llnl.gov/TB) using CrysTool software (2) interfaced with Packard MultiProbeII liquid handling robots.

**RESULTS**. We have performed preliminary studies to optimize steps in small-scale IVT screening. We have compared *Escherichia. coli* extracts from several different vendors. Using the extract of choice, we have obtained similar protein yields using plasmid and PCR template DNA. We have scaled reactions down to 12 µl volumes, which results in significant cost savings in both RTS extracts and FluoroTect™ Green$_{Lys}$. Finally, we have optimized the ratio of labeled to unlabeled lysine in the reactions. The yields from *in vitro* and *in vivo* protein expression correlate well, which validates the use of IVT for expression screening. For large-scale expression, the cDNAs are subcloned into a modified version of pETBlue-2 (Novagen) for high-level expression of C-terminally His-tagged proteins.

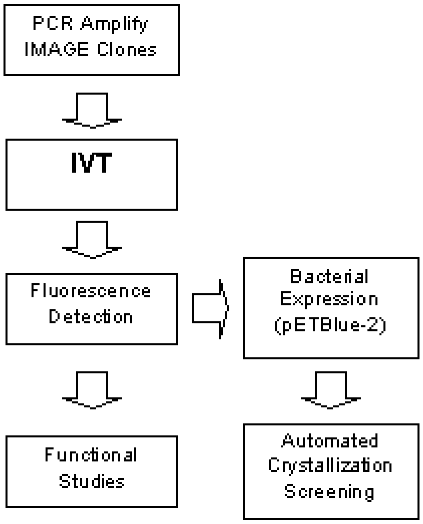

**Figure 1.** See text for details.

**DISCUSSION**. Our IVT system identifies genes, such as those in the IMAGE cDNA collection, that express full-length proteins at significant levels. Our expression screen requires no plasmid preparation or subcloning. Portability to a bacterial expression system allows large-scale production of affinity-tagged proteins for structural studies.

**ACKNOWLEDGEMENTS**. This work was performed under the auspices of the U.S. Dept. of Energy by the University of California, Lawrence Livermore National Laboratory under Contract No. W-7405-Eng-48.

**REFERENCES**

1. Lennon, G.G., Auffray, C., Polymeropoulos, M., Soares, M.B. (1996) Genomics 33, 151–152.
2. Segelke, B.W. (2001) J. Crystal Growth 232, 553–562.
